# Major depression recurrence is associated with differences in obesity-related traits in women, but not in men

**DOI:** 10.1192/j.eurpsy.2024.1764

**Published:** 2024-09-20

**Authors:** Urs Bannert, Ulrike Siewert-Markus, Johanna Klinger-König, Hans J. Grabe, Sylvia Stracke, Marcus Dörr, Henry Völzke, Marcello R.P. Markus, Philipp Töpfer, Till Ittermann

**Affiliations:** 1 University Medicine Greifswald, Greifswald, Germany; 2 Department of Psychiatry and Psychotherapy, University Medicine Greifswald, Greifswald, Germany; 3 German Center for Neurodegenerative Diseases (DZNE), Site, Rostock/Greifswald, Germany; 4 Department of Internal Medicine A, University Medicine Greifswald, Greifswald, Germany; 5 Department of Internal Medicine B, University Medicine Greifswald, Greifswald, Germany; 6 German Centre for Cardiovascular Research (DZHK), Partner Site Greifswald, Greifswald, Germany; 7 Department of Study of Health in Pomerania/Clinical-Epidemiological Research, Institute for Community Medicine, University Medicine Greifswald, Greifswald, Germany; 8 German Center for Diabetes Research (DZD), Partner Site Greifswald, Greifswald, Germany

**Keywords:** cardiometabolic health, comorbidity, major depression, obesity, sex differences

## Abstract

**Background:**

Obesity-related cardiometabolic comorbidity is common in major depressive disorder (MDD). However, sex differences and MDD recurrence may modify the MDD-obesity-link.

**Methods:**

Sex-specific associations of MDD recurrence (single [MDD_S_] or recurrent episodes [MDD_R_]) and obesity-related traits were analyzed in 4.100 adults (51.6% women) from a cross-sectional population-based cohort in Germany (SHIP-Trend-0). DSM-IV-based lifetime MDD diagnoses and MDD recurrence status were obtained through diagnostic interviews. Obesity-related outcomes included anthropometrics (weight, body mass index, waist- and hip-circumference, waist-to-hip ratio, waist-to-height ratio), bioelectrical impedance analysis of body fat mass and fat-free mass, and subcutaneous (SAT) and visceral adipose tissue (VAT) from abdominal magnetic resonance imaging. Sex-stratified linear regression models predicting obesity-related traits from MDD recurrence status were adjusted for age, education, and current depressive symptoms.

**Results:**

790 participants (19.3%) fulfilled lifetime MDD criteria (23.8% women vs. 14.5% men, p<0.001). In women, MDD_S_ was inversely associated with anthropometric indicators of general and central obesity, while MDD_R_ was positively associated with all obesity-related traits, except waist-to-hip ratio and fat-free mass. In women, MDD_R_ versus MDD_S_ was associated with higher levels of obesity across all outcomes except fat-free mass. In men, MDD was positively associated with SAT regardless of MDD recurrence. Additionally, lifetime MDD was positively associated with VAT in men. Results remained significant in sensitivity analyses after exclusion of participants with current use of antidepressants.

**Conclusions:**

The MDD-obesity association is modified by MDD recurrence and sex independent of current depressive symptoms. Accounting for sex and MDD recurrence may identify individuals with MDD at increased cardiometabolic risk.

## Introduction

With an estimated lifetime prevalence of approximately 20% [1], major depressive disorder (MDD) is a common mental disorder. MDD ranks among the leading causes of global disease burden [1, 2] and is associated with worse prognosis and excess mortality risk in various somatic diseases such as cardiovascular diseases (CVDs) and type-2 diabetes [3, 4]. Overweight and obesity, which show reciprocal links with MDD [5], are themselves growing major public health concerns [6]. They represent well-established cardiometabolic risk factors and are associated with increased morbidity and all-cause mortality risk, mainly due to CVD-related deaths [7]. Thus, a significant proportion of disease burden in the context of MDD may be related to the increased cardiometabolic risk associated with obesity in patients with MDD. It is therefore crucial to better understand their complex relationship. In this effort, it should be acknowledged that both MDD and obesity are heterogeneous phenotypes that share common molecular, neural, pathophysiological, and behavioral pathways [8], all of which exhibit significant sex differences [9–15].

With respect to fat metabolism, storage, and distribution, men and women exhibit sexually dimorphic traits [14]. Specifically, women compared to men have more body fat mass (FM), less fat-free mass (FFM) [16], more subcutaneous adipose tissue (SAT), and less visceral adipose tissue (VAT) [14]. Moreover, MDD affects women nearly twice as often as men [17] and with greater symptom severity, including increased appetite and weight gain compared to men [9, 15]. Additionally, subtypes of MDD, such as atypical MDD and MDD recurrence status (MDD_R_), have been associated with weight gain and increased risk of obesity [18, 19], particularly in women [20, 21]. Support for these sex-specific associations was recently provided by a genome-wide association study (GWAS) [12]. Sex-specific polygenic risk for MDD correlates with polygenic risk for obesity-related cardiometabolic risk factors (i.e., body fat, waist-circumference [WC], waist-to-hip ratio [WHR], and triglycerides) in women, but not in men [12].

Thus, it is crucial to adequately consider the above-mentioned evidence on heterogeneity and sex differences in MDD, obesity-related phenotypes, and their co-occurrence to achieve a more precise prediction of cardiometabolic risk in people with MDD. Following recent methodological recommendations for the analyses of sexually dimorphic traits (such as anthropometrics and body fat distribution) [22], we therefore performed sex-stratified analyses to test the sex-specific associations of lifetime MDD, single episode MDD (MDD_s_), and MDD_R_ with anthropometric indicators of general (i.e., weight, body mass index [BMI]) and central obesity (i.e., WC, hip circumference [HC], WHR, waist-to-height ratio [WHtR]) and direct measurements of body fat by magnetic resonance imaging (i.e., VAT, SAT) and bioelectrical impedance (i.e., FM, FFM) in a large, population-based cohort in Northern Germany, the Study of Health in Pomerania (SHIP-Trend-0) [23].

## Method

### Study population

The present cross-sectional study is based on data from the population-based SHIP which was conducted in West Pomerania, the northeastern region of Germany. The study design and recruitment strategy have been described elsewhere [23]. Our analyses are based on data obtained from the second independent cohort (SHIP-TREND-0). At the beginning of 2008, an age- and sex-stratified random sample of 8,826 adults aged 20–79 years was selected from the general population. In total, 4,420 subjects participated in SHIP-TREND-0 between 2008 and 2012 (response rate 50.1%). All participants provided written informed consent. The study was approved by the Local Ethics Committee of the University of Greifswald and followed the Declaration of Helsinki.

Of the 4420 individuals examined in SHIP-TREND-0, we excluded n=320 individuals with missing data in educational status, depression variables, or anthropometric measurements, resulting in a study population of 4,100 individuals (2,115 women, 51.6%) aged 20–83 years. Among them, 3,982 subjects (2,056 women; 51.6%) aged 20–83 years had plausible bioelectrical impedance analysis (BIA) data, and 1,917 individuals (991 women, 51.7%) aged 21–82 years underwent abdominal MRI examination (Supplementary Figure S1).

### Covariates

In a computer-assisted personal interview, the participants stated sociodemographic information about their age, sex (men/women), and years of school education, among others. A categorical variable was used for education indicating low (<10 years), medium (10 years), and high (>10 years) levels of school education. Information on health-related behavior, that is, smoking, alcohol consumption, and physical activity were obtained through self-reports during the interview. More specifically, participants were asked about their current smoking status (never, former, current smoker), and the average alcohol consumption over the past 30 days (in grams per day, g/d) was calculated by multiplying the frequency and amount of alcohol from beer, wine, and spirits, respectively, using a standard ethanol content of 4.8 vol% in beer, 11 vol% in wine, and 33 vol% in spirits for conversion [24]. Individuals were classified as physically active if they reported more than one hour/week of exercise during summer and winter.

### Major depressive disorder

For diagnostic evaluation of MDD, a computerized version of the Munich-Composite International Diagnostic Interview (M-CIDI) [25], a standardized, fully structured instrument to assess psychiatric disorders according to the Diagnostic and Statistical Manual of Mental Disorders (4th Edition; DSM-IV) criteria [26], was administered by experienced clinical psychologists during face-to-face sessions between 2008 and 2012 [27]. Each participant had one single face-to-face session during which the M-CIDI was administered. A participant was classified as positive for lifetime MDD if he or she fulfilled the DSM-IV-based criteria [26] for MDD *at least once* in his or her lifetime. Based on the number of MDD episodes, which were also assessed, participants with a lifetime history of MDD could be further classified as positive for either single episode MDD (i.e., MDD_s_) or recurrent MDD (i.e., MDD_R_).

### Current depressive symptoms

Depressive symptoms during the past 2 weeks were assessed through participants’ self-reports using the Beck Depression Inventory-II (BDI-II) [28]. The BDI-II summary score was calculated using higher scores indicating more severe depressive symptoms.

### Antidepressant medication

All participants were asked to bring all medication taken 7 days prior to the time of examination. Medication data were obtained online using the IDOM program (online drug-database leaded medication assessment) and categorized according to the Anatomical Therapeutical Chemical (ATC) classification index. Antidepressants were defined according to the ATC-code N06A.

### Anthropometric measurements

Part of the regular study protocol of SHIP-Trend-0 were manual anthropometric measurements, including height, weight, and WC and HC, performed by trained study nurses according to a standard operating procedure. The procedure is described in detail in the Supplementary Material.

### Bioelectrical Impedance Analysis (BIA) of fat mass (FM) and fat-free mass (FFM)

To calculate the absolute FM and FFM (in kg) by BIA, a multifrequency Nutriguard‐M device (Data Input, Pöcking, Germany) and the NUTRI4 software (Data Input, Pöcking, Germany) were used in participants without pacemakers. For this purpose, electrodes were placed on the wrist, ankle, and foot. The test frequency was measured at 5, 50, and 100 kHz following the manufacturer’s instructions [29].

### MRI assessment of VAT and SAT

All participants underwent a standardized whole-body MRI examination, which was performed on a 1.5-Tesla MR system (Magnetom Avanto, Siemens Healthcare AG, software version syngo MR B15), to assess body fat distribution (VAT and SAT). All examinations were performed by two trained technicians in a standardized manner. Further details of image acquisition, data analysis, and quality control are provided in the Supplementary Material.

### Statistical analyses

Sex-stratified characteristics of the study population are presented as median, 25th, and 75th percentile for continuous variables and as absolute numbers and percentages for categorical variables. Tests for significant sex differences are based on Kruskal–Wallis tests for continuous variables and the chi-squared test for categorical variables, respectively. Lifetime MDD, MDD_S_, or MDD_R_ were entered as dichotomous categorical predictors in sex-stratified linear regression models adjusted for age, school education, and current depressive symptoms (BDI-II) to predict obesity-related traits. Additional analyses were performed to consider further health-related behaviors, including smoking, alcohol consumption, and physical activity. Models for FFM as an outcome were further adjusted for FM. To exclude the possibility that the results of MDD-obesity associations may be confounded by intake of antidepressants, we performed sensitivity analyses applying identical sex-stratified linear regression models (see above) after exclusion of all participants with current intake of antidepressants (n=217; 69,6% women).

A p<0.05 was considered as statistically significant. All analyses were conducted with Stata 17.0 (Stata Corporation, TX, USA).

## Results

The final analytical sample consisted of 4,100 individuals. Sex-stratified descriptive data for sociodemographic variables, anthropometrics, BIA, and MRI measures, and the prevalence of lifetime MDD diagnosis and MDD recurrence status (MDD_S_, MDD_R_) are shown in Table 1. Women represented 51.6% of the total sample (Table 1) and compared to men had a higher lifetime prevalence for MDD (23.8% vs. 14.5%; p<.001). Among 287 men with lifetime MDD, 109 (5.5%) were diagnosed with MDD_S_ and 178 (9.0%) with MDD_R_. Among 503 women with lifetime MDD, 136 (6.4%) had a diagnosis of MDD_S_, while 357 (16.9%) had MDD_R_. A total of 217 individuals (66 men, 151 women, p<0.001) currently took antidepressants. Significant sex differences were observed for all anthropometric markers and measures of body composition and fat distribution (all p<0.001), except HC (p=0.657) (Table 1).

**Table 1.** Characteristics of the study population stratified by sex (N=4100)

	MenN=1,985 (48.4%)	WomenN=2,115 (51.6%)	*p*-value^a^
Age; years	52 (40; 64)	51 (39; 62)	0.008
Level of education (%)			<0.001
Low (<10 years)	465 (23.4%)	424 (20.0%)	
Medium (10 years)	953 (48.0%)	1,198 (56.6%)	
High (>10 years)	567 (28.6%)	493 (23.3%)	
Smoking status			<0.001
Never	518 (26.1%)	978 (46.2%)	
Former	884 (44.5%)	605 (28.6%)	
Current	583 (29.4%)	532 (25.2%)	
Body height; cm	177 (172; 181)	164 (159; 169)	<0.001
Body weight; kg	87.2 (78.3; 97.8)	71.6 (63.2; 81.9)	<0.001
Body mass index; kg/m^b^	28.1 (25.5; 31.1)	26.6 (23.3; 30.7)	<0.001
Waist circumference; cm	97 (88; 105)	83 (75; 93)	<0.001
Hip circumference; cm	101 (97; 107)	101 (94; 110)	0.657
Waist-to-hip ratio	0.95 (0.90; 0.99)	0.82 (0.78; 0.86)	<0.001
Waist-to-height ratio	0.55 (0.49; 0.60)	0.51 (0.45; 0.58)	<0.001
Fat mass; kg	20.8 (16.2; 26.0)	24.5 (18.4; 31.8)	<0.001
Fat-free mass; kg	66.7 (61.2; 72.6)	47.2 (44.0; 51.3)	<0.001
Subcutaneous fat; L	6.5 (4.9; 8.5)	8.3 (6.2; 11.1)	<0.001
Visceral fat; L	5.2 (3.1; 7.1)	2.5 (1.3; 3.9)	<0.001
MDD (lifetime)	287 (14.5%)	503 (23.8%)	<0.001
MDD_S_	109 (5.5%)	136 (6.4%)	0.024
MDD_R_	178 (9.0%)	357 (16.9%)	<0.001
Current depressive symptoms (BDI–2)	5.48 (3.19 9.33)	7.17 (4.38 11.12)	<0.001
Intake of antidepressants	66 (3.3%)	151 (7.1%)	<0.001
Associated with weight gain^b^	20 (1.0%)	53 (2.5%)	<0.001
Associated with weight reduction^b^	5 (0.3%)	5 (0.2%)	0.920
Other antidepressants^c^	41 (2%)	93 (4.4%)	<0.001
Sleeping disorders	308 (15.5%)	553 (26.2%)	<0.001
Ethanol in g/d within the last 30 days	8.0 (2.1; 18.5)	1.8 (0.4; 5.0)	<0.001
Regular physical activity, winter, and summer	938 (47.3%)	984 (46.5%)	0.640

Abbreviations: BDI-2, Beck Depression Inventory-2. MDD, major depressive disorder; MDD_R_, recurrent MDD; MDD_S,_ single episode MDD.

*Note*: Data are expressed as median, 25th and 75th percentile (continuous data) or in absolute numbers and percentage of sex-stratified subgroups (categorical data).

a *p*-values are based on the chi-squared test for categorical variables and the Kruskal-Wallis tests for continuous variables [41, 42].

b Antidepressants with meta-analytical evidence for association with weight gain (i.e., Amitryptilin [ATC N06AA09], Mirtazapin [ATC N06AX11], Paroxetin [ATC N06AB05]) or weight reduction (i.e., Fluoxetin [N06AB03], Bupropion [ATC N06AX12]) [36, 37].

c Includes all pharmacological agents classified under ATC code N06A.

## Sex-specific associations of lifetime MDD with obesity-related traits

**Figure 1.**
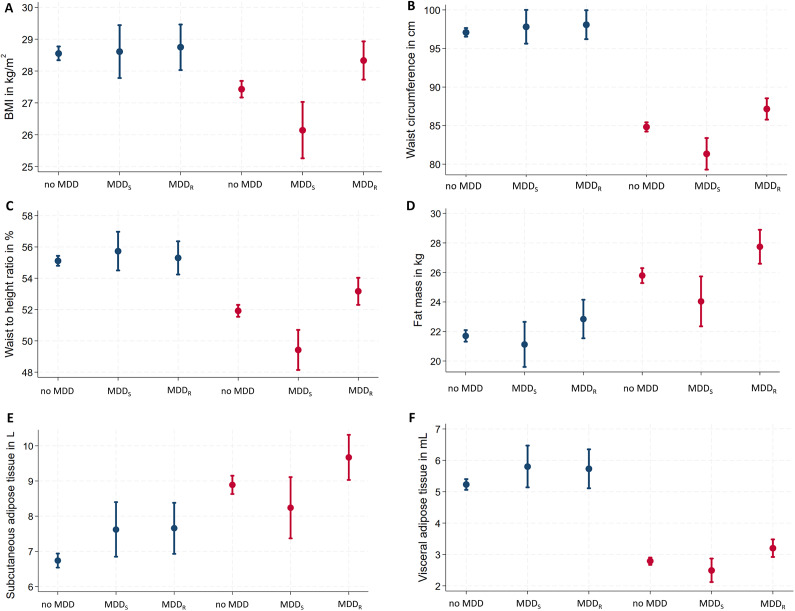
Sex-specific associations of MDD recurrence and obesity-related traits. Data are presented as adjusted means (95% confidence interval)* for (A) BMI, (B) waist circumference, (C) waist-to-height ratio, (D) fat mass, (E) subcutaneous adipose tissue, and (F) visceral adipose tissue. *Linear regression models are adjusted for age, education, and current depressive symptoms. BMI, body mass index; MDD, major depressive disorder; MDD_R,_ recurrent major depressive disorder; MDD_S_, single episode major depressive disorder.

Adjusting for age, education, and current depressive symptoms, sex-stratified regression models for the associations between lifetime MDD, anthropometric outcomes, and body fat revealed a sex-specific pattern of results, as shown in Table 2 and Figure 1.

**Table 2.** Sex-specific associations between MDD subtypes and obesity-related traits

	MDD lifetime vs. no MDDβ (95%-CI)	MDD_S_ vs. no MDDβ (95%-CI)	MDD_R_ vs. no MDDβ (95%-CI)	MDD_R_ vs. MDD_S_β (95%-CI)
Men				
Body weight (kg)	0.76 (−1.29; 2.82)	−0.49 (−3.43; 2.45)	1.71 (−0.88; 4.30)	2.20 (−1.48; 5.87)
BMI (kg/m^2^)	0.14 (−0.47; 0.74)	0.06 (−0.81; 0.92)	0.20 (−0.56; 0.96)	0.14 (−0.94; 1.22)
WC (cm)	0.87 (−0.71; 2.45)	0.72 (−1.54; 2.98)	0.99 (−1.01; 2.98)	0.27 (−2.56; 3.10)
HC (cm)	0.08 (−1.10; 1.27)	−0.09 (−1.79; 1.61)	0.21 (−1.28; 1.71)	0.30 (−1.82; 2.43)
WHR	0.77 (−0.11; 1.65)	0.86 (−0.39; 2.11)	0.70 (−0.40; 1.81)	−0.16 (−1.72; 1.41)
WHtR	0.37 (−0.53; 1.27)	0.62 (−0.66; 1.90)	0.19 (−0.94; 1.31)	−0.43 (−2.03; 1.17)
Fat mass (kg)	0.32 (−0.78; 1.42)	−0.58 (−2.16; 1.00)	1.14 (−0.24; 2.52)	1.60 (−0.38; 3.58)
Fat-free mass (kg)	−0.21 (−1.17; 0.74)	−0.01 (−1.38; 1.35)	−0.36 (−1.57; 0.84)	−0.35 (−2.06; 1.37)
SAT (L)	0.90 (0.31; 1.50)**	0.89 (0.08; 1.69)*	0.92 (0.16; 1.69)*	0.04 (−0.99; 1.07)
VAT(L)	0.54 (0.03; 1.04)*	0.58 (−0.11; 1.26)	0.50 (−0.15; 1.16)	−0.07 (−0.96; 0.81)
Women				
Body weight (kg)	1.28 (−0.33; 2.90)	−2.32 (−4.90; 0.27)	2.94 (1.06; 4.83)**	5.26 (2.27; 8.27)**
BMI (kg/m^2^)	0.21 (−0.37; 0.79)	−1.29 (−2.21; −0.36)**	0.91 (0.23; 1.58)**	2.19 (1.12; 3.26)***
WC (cm)	0.49 (−0.85; 1.82)	−3.49 (−5.62; −1.35)**	2.33 (0.77; 3.89)**	5.82 (3.35; 8.29)***
HC (cm)	0.70 (−0.52; 1.94)	−1.85 (−3.81; 0.11)	1.87 (0.44; 3.30)*	3.72 (1.45; 5.99)**
WHR	−0.09 (−0.76; 0.59)	−1.88 (−2.94; −0.80)**	0.76 (−0.02; 1.54)	2.64 (1.40; 3.88)***
WHtR	0.06 (−0.78; 0.90)	−2.49 (−3.83; −1.16)***	1.25 (0.27; 2.22)*	3.75 (2.20; 5.29)***
Fat mass (kg)	0.77 (−0.34; 1.88)	−1.75 (−3.51; 0.02)	2.87 (1.72; 4.02)**	3.85 (1.80; 5.90)***
Fat-free mass (kg)	−0.11 (−0.32; 0.54)	0.07 (−0.61; 0.75)	0.11 (−0.39; 0.61)	0.04 (−0.75; 0.83)
SAT (L)	0.26 (−0.33; 0.86)	−0.65 (−1.56; 0.26)	0.78 (0.07; 1.49)*	1.43 (0.35; 2.51)*
VAT(L)	0.15 (−0.10; 0.41)	−0.30 (−0.69; 0.10)	0.41 (0.10; 0.72)**	0.71 (0.24; 1.17)**

Abbreviations: BDI-2, Beck Depression Inventory 2; BMI, body mass index; CI, confidence interval; HC, hip circumference; MDD, major depressive disorder; MDD_R_, recurrent MDD; MDD_S_, single episode MDD; SAT, subcutaneous adipose tissue; VAT, visceral adipose tissue; WC, waist circumference; WHR, waist-to-hip ratio; WHtR, Waist-to-height ratio.

*Note*: β coefficients are derived from sex-stratified linear regression models adjusted for age, education, and current depressive symptoms (BDI-2). Models for fat-free mass as outcome are further adjusted for fat mass.

β coefficients marked in bold are significant at *p<0.05, **p<.01, ***p<.001, respectively.

In men, lifetime MDD showed positive associations with SAT and VAT. In contrast, lifetime MDD in women exhibited no significant positive association for any anthropometric or body fat marker. Excluding participants with current antidepressants intake did not change the results (Supplementary Table S1). In regression models for lifetime MDD, independent positive effects of current depressive symptoms were observed for all obesity-related traits in women but only for WHtR in men (Supplementary Table S2).

## Sex-specific associations of MDD recurrence status with obesity-related traits

In men, both MDD_S_ and MDD_R_ were associated with increased SAT. No other significant associations were observed between MDD recurrence status and any other anthropometric or body fat marker (Table 2). Among men with lifetime MDD, no differences in any obesity-related trait emerged between men with MDD_S_ versus MDD_R_. Excluding participants with current antidepressants intake did not change the results (Supplementary Table S1) or the inclusion of additional covariates (Supplementary Table S4).

In women, MDD_S_ was associated with decreased BMI, WC, WHR, and WHtR (Table 2). In contrast, MDD_R_ exhibited significant positive associations with all anthropometric and body fat measures, except WHR and FFM. Specifically, MDD_R_ was positively associated with body weight, BMI, WC, HC, WHtR, FM, SAT, and VAT (Table 2). Among women with lifetime MDD, significant differences associated with MDD recurrence status were observed for all obesity-related traits, except FFM (Table 2) with consistently increased obesity-related traits in women with MDD_R_. Excluding participants with current antidepressants intake, associations of MDD_R_ with BMI and WHtR were no longer significant in women. Differences related to MDD recurrence status remained significant for all obesity-related traits, except FFM (Supplementary Table S1). Inclusion of additional covariates (smoking, alcohol consumption, physical activity) only marginally altered the associations but did not change the pattern of the results (Supplementary Table S4).

## Sex-specific associations of antidepressant medication with obesity-related traits

Secondary analyses revealed that after adjusting for age and education, current intake of antidepressants showed an overall positive association with obesity-related traits in men and women (Supplementary Table S3). Across obesity-related traits, effects related to current antidepressant intake were more consistent and larger in women compared to men (Supplementary Table S3).

## Discussion

The aim of the present study was to explore sex-specific associations of lifetime MDD and MDD recurrence status (i.e., MDD_S_, vs. MDD_R_) with obesity-related traits in an adult community sample. Specifically, the associations between MDD recurrence status and obesity-related traits revealed a consistent contrary pattern in women, but not in men. In women, MDD_S_ showed significant *inverse* associations with general (i.e., BMI) and central obesity (i.e., WC, WHR, and WHtR), while MDD_R_ exhibited significant *positive* associations with body weight; general (BMI) and central obesity (WC, HC, WHtR); and markers of body fat (FM, SAT, and VAT). Of note, only MDD recurrence status but not lifetime MDD showed significant associations with obesity-related traits in women. In men, just a few effects of lifetime MDD and MDD recurrence status on obesity-related traits were observed and limited to increases in SAT and VAT.

Consistent with established sex differences in lifetime prevalence [30], women in our cohort were at greater risk for lifetime MDD compared to men (23.8% vs. 14.5%), largely due to sex differences in MDD_R_ prevalence (16.9% vs. 9.0%). Results are further consistent with evidence linking MDD to anthropometric markers of obesity and weight gain in women [15, 20, 31], which may partly be driven by women with MDD_R_ [21]. However, other studies did not report evidence of an effect modification by sex, MDD recurrence status, or their interaction [5]. Data on sex-specific links between MDD and direct measures of body fat are scarce and inconclusive, likely due to small sample sizes [32]. Previous population-based studies did not investigate the sex-specific effects of MDD or MDD recurrence status, but revealed positive associations between current depressive symptoms and VAT in women [33, 34], men [35], and both sexes [36]. We included current depressive symptoms (BDI-2 scores) in all MDD regression models and were able to show that, independent of lifetime MDD, current depressive symptoms exhibited significant positive associations with all obesity-related traits (except FFM) in women (Supplementary Table S2). In men, only the WHtR showed a positive relationship with current depressive symptoms. Importantly, the observed effects of MDD_S_ and MDD_R_ in our study were independent of current depressive symptoms. Thus, MDD recurrence status may exhibit distinct patterns of obesity-related traits in women, irrespective of current mood. We are not aware of any other population-based study investigating associations between MDD, MDD recurrence status, and body fat markers that simultaneously accounted for sex differences and MDD heterogeneity. It is particularly worth noting that our results suggest that women with MDD_R_ exhibit a pattern of obesity-related traits that is reminiscent of patients with “atypical” MDD, an MDD subtype which among other symptoms is characterized by hyperphagia (i.e., increased appetite). Indeed, epidemiological data suggest that, on average, women are two to three times more likely than men to present with symptoms consistent with the “atypical” MDD subtype [37, 38]. Additionally, and not taking the “atypical” MDD subtype into account, recent evidence from a GWAS suggests a female-specific link in the genetic association of MDD and obesity-related traits [39]. These data converge to suggest that “atypical” MDD may constitute an MDD phenotype that is more characteristic for women than men [15, 21, 39]. We would further like to highlight that our results (summarized in Table S2) clearly show that current depressive symptoms (BDI-2), without taking the subtype of “atypical” MDD into account, show a clear pattern of sex-specific associations. Across all outcomes, depressive symptoms are significantly and positively associated with obesity-related traits in women, whereas in men only WHtR reveals a modest positive association. We (and others) therefore consider the label “atypical” problematic in this context. “Atypical” syndromes almost always refer to disease phenotypes that are more frequent in or more “typical” for women. This likely reflects a historical trend where males were largely overrepresented in clinical research and therefore defined the normative clinical presentation for most disease entities.

Given the well-documented obesogenic effects of antidepressants [40–42], we conducted sensitivity analyses to exclude the possibility of confounding by the current intake of antidepressants. In participants without current antidepressant medication, most results remained unchanged (Supplementary Table S1) compared to the total sample. Of note, in women, associations of MDD_R_ with BMI and WHtR were no longer significant in sensitivity analyses, suggesting a potential mediation effect of antidepressants between MDD_R_ and anthropometric markers of obesity in women. Consistent with previous reports [40–42], exploratory analyses in participants with current antidepressant intake revealed strong links of antidepressants with obesity-related traits (Supplementary Table S3), which were more pronounced in women than men, a finding that is consistent with previously published data on this topic [43]. This finding further corroborates evidence indicating that the adverse effects of pharmacological agents, including antidepressants, disproportionately affect women [44]. Furthermore, the frequent off-label use of antidepressants for other health problems, such as pain and sleep disorders and the rapid incline of prescription of antidepressants [45] warrants careful consideration of the adverse drug reactions related to cardiometabolic health, with an emphasis on potential sex differences. Given the relatively small number of medicated participants, caution is warranted in drawing premature conclusions though. Nevertheless, we propose a systematic investigation of this effect in randomized controlled clinical trials, well-powered clinical trials, or population-based cohorts.

Given the growing public health challenges associated with rising MDD and obesity levels, and their associated cardiometabolic health risks in the general population, there is an urgent need to better understand their mechanistic links. Several excellent reviews [8, 46] provide insight into possible behavioral, molecular, neurobiological, neuroendocrine, and immunological pathways at the intersection of MDD and obesity-related traits, although sex and gender aspects are not systematically considered in this context. Meta-analytical evidence indicates that women with MDD are more likely than men with MDD to report increased appetite and subsequent weight gain [9]. Considering sex (but not MDD heterogeneity), a recent GWAS in a large predominantly white European sample revealed a sex-specific polygenic risk for MDD [12]. Importantly, sex-specific polygenic MDD risk was associated with polygenic risk for obesity-related traits and metabolic features (i.e., body fat, WC, WHR, and triglycerides) in women, but not in men. Silveira et al. [12] further identified expression regulation of the dopamine-receptor-D_2_ gene (*DRD2*) as a depression-associated mechanism in women only. Given that deficient dopaminergic signaling has been identified in patients with disordered eating [47], obesity [48], and MDD [49], all of which occur more frequently in women, we speculate that this (genetically regulated) dopaminergic pathway may represent a female-biased mechanism linking MDD and obesity, particularly in MDD_R_.

There are further plausible candidate mechanisms associated with cardiometabolic risk that exhibit significant sex differences and have well-established roles in both MDD and obesity [8, 46]. For instance, both inflammation and dysregulation of the hypothalamic-pituitary-adrenal (HPA) axis have been associated with MDD [50, 51] and obesity [52, 53]. While both the immune system [10] and the HPA axis [13] show remarkable sex differences, their sex-differential roles in MDD, obesity, and their co-occurrence remains inconclusive. This lack of evidence warrants further systematic investigation of sex differences and additional factors that may interact with sex to predict differences in disease mechanisms relevant to MDD and obesity-related comorbidity which may (among others) include age, hormonal status (e.g., pregnancy, menopause), and exposure to childhood maltreatment.

There are several limitations in our analyses that we need to acknowledge. First, the cross-sectional observational study design does not permit the inference of causal pathways or longitudinal associations, which limits our ability to establish mechanistic or prospective associations (e.g., changes in weight and/or appetite) in the MDD-obesity relationship, which were mainly observed in women. Moreover, we cannot exclude the possibility of residual confounding by factors that we have not considered in statistical models or were not measured. As another limitation, our cohort is ethnically highly homogenous. All participants were white German nationals from a northeastern region of Germany (Western Pomerania). This circumstance limits the generalizability of our study to other populations of different ethnic, racial, cultural, or geographical background, where associations of MDD and obesity may systematically differ from the SHIP cohort.

A major strength of our study is the fact that in addition to standard anthropometric markers, we obtained direct measures of body fat through BIA and MRI in a large community-based sample. Given that body fat tissue, especially VAT, is associated with increased cardiometabolic risk in obese individuals [54], the present study may provide important novel insights how MDD could contribute to an increased risk for chronic diseases in both men and women. As another strength, MDD diagnoses were established by trained clinicians in face-to-face sessions using structured clinical interviews and current depressive symptoms were accounted for in statistical models suggesting that MDD recurrence status and current depressive symptoms may be considered independent sex-specific predictors of obesity-related traits. Moreover, our stratification strategy accounts for sex and MDD heterogeneity and identified previously unreported associations, e.g., decreased general and central obesity in women with MDD_S_. DSM-5-based criteria for MDD diagnosis include weight loss/gain and decreased or increased appetite [55]. Adequate stratification of MDD patients (e.g., based on MDD recurrence status) may help to establish greater diagnostic accuracy for subsamples of MDD patients with important treatment implications. For instance, inflammation is a hallmark feature of obesity and is associated with reduced treatment response to antidepressants [56]. Evidence from this study and others further suggests that MDD [15, 20], MDD recurrence status [21], and antidepressants [40, 41] are associated with higher obesity risk, possibly more so in women. Together, these factors may contribute to increased cardiometabolic risk and reduced pharmacological treatment efficacy in subgroups of MDD patients and careful consideration of these issues may help to improve treatment outcomes, particularly for patients with MDD and obesity.

To conclude, by considering sex differences and MDD heterogeneity, we provide novel epidemiological insights into the intricate MDD–obesity relationship, which may help identify individuals with MDD at increased cardiometabolic risk associated with obesity.

## Supporting information

Bannert et al. supplementary material Bannert et al. supplementary material

## Data Availability

Data from the “Study of Health in Pomerania” are available from the University Medicine Greifswald, Germany but restrictions apply to the availability of these data, which were used under license for the current study, and so are not publicly available. Data are, however, available upon reasonable request at https://transfer.ship-med.uni-greifswald.de/FAIRequest/data-use-intro and with permission of the University Medicine Greifswald.

## Supplementary material

The supplementary material for this article can be found at http://doi.org/10.1192/j.eurpsy.2024.1764.
