# Supplementary material for: Major depression recurrence is associated with differences in obesity-related traits in women, but not in men
Source: Eur Psychiatry. 2024 Sep 20;67(1):e55. doi: 10.1192/j.eurpsy.2024.1764 (PMC11457113; doi:10.1192/j.eurpsy.2024.1764)
Supplement: Bannert et al. supplementary material [file S0924933824017644sup001.docx]

**Supplemental Material**

**Major Depression Recurrence is Associated with Differences in Obesity-related Traits in Women, but not in Men**

U. Bannert^1^, U. Siewert-Markus, PhD^2^, J. Klinger-König, MSc^2^, H. J. Grabe, MD^2,3^, S. Stracke, MD^4^, M. Dörr, MD^5, 6^, H. Völzke, MD^7^, M. R. P. Markus, MD, PhD^5, 6, 8^, P. Töpfer, PhD^4^* , T. Ittermann, PhD^6, 7^*

* these authors contributed equally.

^1^ University Medicine Greifswald, Germany.

^2^ Department of Psychiatry and Psychotherapy, University Medicine Greifswald, Greifswald, Germany.

^3^ German Center for Neurodegenerative Diseases (DZNE), Site Rostock/Greifswald, Germany

^4^ Department of Internal Medicine A, University Medicine Greifswald, Greifswald, Germany.

^5^ Department of Internal Medicine B, University Medicine Greifswald, Greifswald, Germany.

^6^ German Centre for Cardiovascular Research (DZHK), partner site Greifswald, Greifswald, Germany.

^7^ Department of Study of Health in Pomerania/Clinical-Epidemiological Research, Institute for Community Medicine, University Medicine Greifswald, Greifswald, Germany.

^8^ German Center for Diabetes Research (DZD) partner site Greifswald, Greifswald, Germany.

**Supplemental Methods**

Anthropometric measurements: Weight was measured to the nearest 0.1 kg in light clothing and without shoes using standard digital scales. Body height was measured to the nearest 0.1 cm using a portable stadiometer (Soehnle Industrial Solutions, Backnang, Germany). Based on these measurements, BMI was calculated using the standard formula (BMI = weight (kg)/height^2^ (m^2^)). Waist and hip circumference were measured with a flexible, non-stretchable graduated tape recommended by the World Health Organization ^1^. Each participant received exact instructions to stand upright with a study nurse standing in a lateral position behind the participant. For the measurement of waist circumference, the examiner palpated the iliac crest and the lowest rip at the lateral part of the body, and the final measurement was made in the middle of these two landmarks. To assess hip circumference, the examiner palpated the most lateral point of the greater trochanter and the iliac crest and performed the final measurement in the middle of these reference points. Subsequently, we calculated waist-to-hip ratio (WHR) as well as waist-to-height ratio (WHtR).

Magnetic Resonance Imaging (MRI) assessment of visceral adipose tissue (VAT) and subcutaneous adipose tissue (SAT): Visceral and subcutaneous fat volumes were determined using a two-echo chemical shift-encoded gradient echo sequence in axial orientation acquired by covering the abdomen in three stacks (each with 64 slices) with the following imaging parameters: repetition time: 7.5 ms; echo time: 2.4/4.8 ms; flip angle: 10°; voxel size: 1.64 × 1.64 × 3.0 mm; slice gap: none; field of view: 420 × 288 mm; matrix: 256 × 120; bandwidth: 290 Hz per pixel; and parallel imaging with an effective acceleration factor of 2.0. Data analysis was processed via ‘ATLAS’ (Automatic Tissue Labelling Analysis Software), which was specifically programmed for this purpose.^2^ Afterwards, the results were manually corrected. Within the manual correction, the upper (left diaphragm) and lower margins (bladder) for the abdominal fat analyses were set, misclassified fat labels corrected, and fat labels that did not belong to the abdomen removed (i.e. arms, breast fat, and parenchyma, bone marrow of pelvis and spine). This was performed by certified medical students (intraclass correlation coefficient > 0.997). VAT and SAT are presented as volumes in liters.


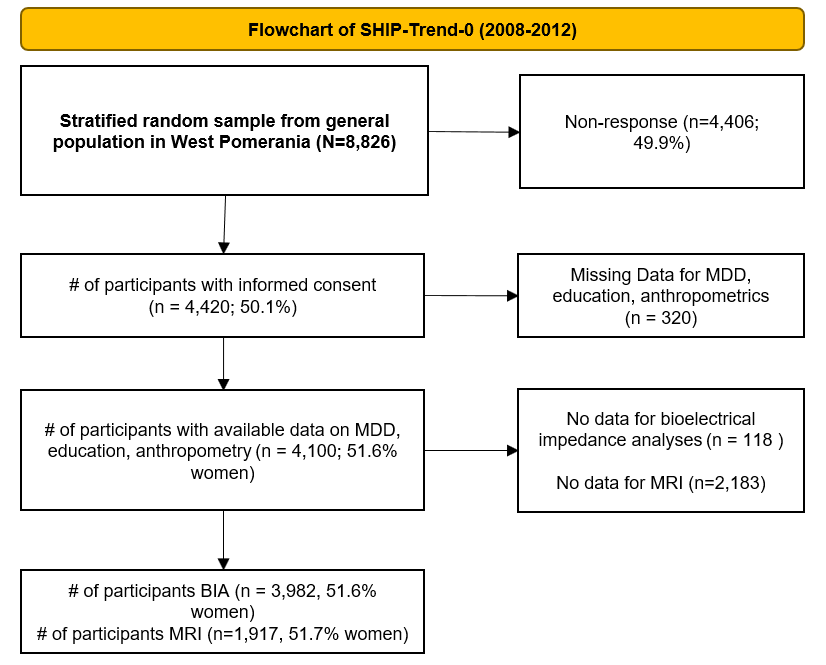


**Figure S1**: Study flow-chart.

**Table S1.** Sex-specific associations between lifetime history of major depression (MDD), MDD subtypes, and obesity-related traits in participants without current intake of antidepressants.^1^

|  | **MDD lifetime vs. no MDD**  **β (95%-CI)** | **MDD_S_ vs. no MDD**  **β (95%-CI)** | **MDD_R_ vs. no MDD**  **β (95%-CI)** | **MDD_R_ vs. MDD_S_**  **β (95%-CI)** |
| --- | --- | --- | --- | --- |
| **Men** | | | | |
| Body weight (kg) | 0.39 (-1.75; 2.53) | -0.06 (-3.07; 2.95) | 0.76 (-3.07; 2.95) | 0.83 (-3.04; 4.69) |
| BMI (kg/m^2^) | 0.14 (-0.49; 0.76) | 0.20 (-0.68; 1.08) | 0.08 (-0.72; 0.89) | -0.12 (-1.25; 1.01) |
| WC (cm) | 0.72 (-0.93; 2.37) | 0.92 (-1.40; 3.25) | 0.55 (-1.58; 2.69) | -0.37 (-3.36; 2.61) |
| HC (cm) | -0.02 (-1.25; 1.22) | 0.10 (-1.64; 1.83) | -0.11 (-1.70; 1.49) | -0.21 (-2.44; 2.02) |
| WHR | 0.70 (-0.22; 1.62) | 0.90 (-0.39; 2.20) | 0.54 (-0.65; 1.73) | -0.37 (-2.03; 1.30) |
| WHtR | 0.40 (-0.53; 1.34) | 0.73 (-0.58; 2.05) | 0.13 (-1.08; 1.34) | -0.60 (-2.29; 1.08) |
| Fat mass (kg) | 0.25 (-0.89; 1.38) | -0.31 (-1.92; 1.30) | 0.89 (-0.58; 2.36) | 1.03 (-1.04; 3.10) |
| Fat-free mass (kg) | -0.41 (-1.41; 0.59) | 0.10 (-1.31; 1.50) | -0.83 (-2.12; 0.47) | -0.92 (-2.74; 0.90) |
| SAT (L) | **0.95 (0.34; 1.56)**** | **0.94 (0.11; 1.76)*** | **0.97 (0.16; 1.77)*** | 0.03 (-1.05; 1.12) |
| VAT(L) | **0.62 (0.10; 1.15)*** | 0.68 (-0.03; 1.40) | 0.57 (-0.13; 1.26) | -0.12 (-1.05; 0.82) |
| **Women** | | | | |
| Body weight (kg) | 0.96 (-0.72; 2.64) | -1.98 (-4.60; 0.64) | **2.45 (0.45; 4.45)*** | **4.43 (1.34; 7.52)**** |
| BMI (kg/m^2^) | 0.07 (-0.54; 0.67) | -**1.18 (-2.11; -0.24)*** | 0.71 (-0.01; 1.42) | **1.88 (0.78; 2.99)**** |
| WC (cm) | 0.13 (-1.27; 1.54) | -**3.40 (-5.59; -1.22)**** | **1.95 (0.28; 3.62)*** | **5.35 (2.77; 7.93)***** |
| HC (cm) | 0.53 (-0.76; 1.81) | -1.60 (-3.60; 0.40) | **1.59 (0.06; 3.12)*** | **3.19 (0.83; 5.56)**** |
| WHR | -0.27 (-0.99; 0.44) | **-1.99 (-3.11; -0.88)***** | 0.63 (-0.22; 1.48) | **2.63 (1.31; 3.94)***** |
| WHtR | -0.17 (-1.05; 0.71) | **-2.45 (-3.82; -1.08)***** | 1.02 (-0.03; 2.06) | **3.47 (1.85; 5.09)***** |
| Fat mass (kg) | 0.56 (-0.59; 1.71) | -1.44 (-3.22; 0.33) | **1.60 (0.23; 2.97)*** | **3.21 (1.10; 5.32)**** |
| Fat-free mass (kg) | 0.11 (-0.34; 0.56) | 0.10 (-0.60; 0.78) | 0.10 (-0.44; 0.64) | 0.01 (-0.82; 0.83) |
| SAT (L) | 0.30 (-0.32; 0.96) | -0.53 (-1.47; 0.41) | **0.80 (0.05; 1.55)*** | **1.33 (0.20; 2.46)*** |
| VAT(L) | 0.18 (-0.09; 0.45) | -0.26 (-0.67; 0.15) | **0.45 (0.12; 0.78)**** | **0.71 (0.22; 1.20)**** |

**Note:** β coefficients are derived from sex-stratified linear regression models adjusted for age, education, and current depressive symptoms (BDI-2). Models for fat-free mass as outcome are further adjusted for fat mass.

β coefficients marked in bold are significant at *p<0.05, **p<.01, ***p<.001, respectively.

^1^ Exclusion of all pharmacological agents classified under ATC code N06A (n=224).

**Abbreviations;** BDI-2 = Beck Depression Inventory 2; BMI = Body mass index; CI = confidence interval; HC = Hip circumference; MDD = major depressive disorder; MDD_R_ = recurrent MDD; MDD_S_ = single episode MDD; SAT = Subcutaneous adipose tissue; VAT = visceral adipose tissue; WC = Waist circumference; WHR = Waist-to-hip ratio; WHtR = Waist-to-height ratio.

**Table S2.** Sex-specific associations between current depressive symptoms and obesity-related traits.

|  | Men | Women |
| --- | --- | --- |
|  | **Depressive symptoms (BDI-2)**  **β (95%-CI)** | **Depressive symptoms (BDI-2)**  **β (95%-CI)** |
| Body weight; kg | 0.06 (-0.06; 0.19) | **0.24 (0.13; 0.35)***** |
| Body mass index; kg/m^2^ | 0.04 (-0.01; 0.07) | **0.09 (0.05; 0.13)***** |
| Waist circumference; cm | 0.08 (-0.01; 0.18) | **0.24 (0.15; 0.33)***** |
| Hip circumference; cm | 0.07 (-0.01; 0.14) | **0.18 (0.09; 0.26)***** |
| Waist to hip ratio*100 | 0.02 (-0.04; 0.07) | **0.09 (0.04; 0.14)***** |
| Waist to height ratio*100 | **0.06 (0.01; 0.12)*** | **0.16 (0.10; 0.21)***** |
| Fat mass; kg | 0.06 (-0.01; 0.12) | **0.18 (0.11; 0.26)***** |
| Fat-free mass; kg | -0.04 (-0.10; 0.02) | **-0.03 (-0.06; -0.01)*** |
| Subcutaneous fat; L | 0.01 (-0.03; 0.05) | **0.06 (0.01; 0.10)*** |
| Visceral fat; L | 0.03 (-0.01; 0.06) | **0.02 (0.01; 0.04)*** |

**Note:** β coefficients for BDI-2 models are derived from sex-stratified linear regression models adjusted for age, education, and lifetime MDD diagnosis. Models for fat-free mass as outcome are further adjusted for fat mass.

β coefficients marked in bold are significant at *p<0.05, **p<.01, ***p<.001, respectively.

**Abbreviations:** BDI-2 = Beck Depression Inventory 2; CI = confidence interval.

**Table S3:** Sex-specific associations of antidepressants with obesity-related traits.

| **Men** | | | |
| --- | --- | --- | --- |
|  | **Antidepressants associated with weight gain**^1^ **(n=20)** | **Other antidepressants (n=41)**^2^ | **Antidepressants combined (n=66)**^3^ |
| Body weight (kg) | 3.81 (-2.81; 10.43) | 1.52 (-0.36; 3.40) | **5.24 (1.55; 8.92)**** |
| BMI (kg/m^2^) | 0.96 (-0.98; 2.91) | 0.11 (-1.19; 1.42) | **1.24 (0.16; 2.32)*** |
| WC (cm) | 4.09 (-1.01; 9.19) | 1.37 (-2.05; 4.80) | **3.99 (1.15; 6.82)**** |
| HC (cm) | 2.43 (-1.40; 6.25) | -0.07 (-2.64; 2.50) | **2.57 (0.44; 4.70)**** |
| WHR | 1.84 (-0.98; 4.66) | 1.25 (-0.64; 3.15) | 1.48 (-0.09; 3.05) |
| WHtR | 2.12 (-0.77; 5.01) | 0.25 (-1.69; 2.19) | **1.76 (0.15; 3.37)*** |
| Fat mass (kg) | 1.23 (-2.36; 4.81) | 1.25 (-1.12; 3.62) | **2.77 (0.80; 4.74)**** |
| Fat-free mass (kg) | 1.26 (-1.86; 4.37) | -0.75 (-2.81; 1.31) | 0.27 (-1.45; 1.99) |
| SAT (L) | 0.96 (-1.15; 3.08) | 0.29 (-1.40; 1.99) | 0.81 (-0.56; 2.17) |
| VAT(L) | 0.14 (-1.67; 1.95) | -0.15 (-1.61; 1.30) | 0.05 (-1.11; 1.23) |
| **Women** | | | |
|  | **Antidepressants associated with weight gain**^1^ **(n=53)** | **Other antidepressants (n=93)**^2^ | **Antidepressants combined (n=151)**^3^ |
| Body weight (kg) | **9.24 (5.22; 13.3)***** | **4.03 (1.04; 7.02)**** | **6.43 (3.99; 8.88)***** |
| BMI (kg/m^2^) | **3.11 (1.67; 4.56)***** | **1.62 (0.55; 2.69)**** | **2.33 (1.45; 3.21)***** |
| WC (cm) | **7.19 (3.86; 10.5)***** | **3.02 (0.54; 5.50)*** | **5.04 (3.01; 7.07)***** |
| HC (cm) | **6.21 (3.15; 9.26)***** | **2.45 (0.18; 4.72)*** | **4.25 (2.40; 6.11)***** |
| WHR | **1.81 (0.14; 3.49)**** | 1.04 (-0.20; 2.29) | **1.45 (0.42; 2.47)**** |
| WHtR | **4.19 (2.10; 6.29)***** | **1.97 (0.41; 3.52)*** | **3.06 (1.79; 4.33)***** |
| Fat mass (kg) | **6.73 (3.99; 9.47)***** | **3.22 (1.12; 5.32)**** | **4.92 (3.22; 6.61)***** |
| Fat-free mass (kg) | -0.42 (-1.47; 0.63) | 0.07 (-0.73; 0.87) | -0.09 (-0.74; 0.57) |
| SAT (L) | **2.00 (0.56; 3.44)*** | 1.02 (-0.16; 2.20) | **1.61 (0.68; 2.54)**** |
| VAT(L) | **0.72 (0.10; 1.34)*** | 0.44 (-0.07; 0.95) | **0.60 (0.20; 1.00)**** |

**Note:** β coefficients are derived from sex-stratified linear regression models adjusted for age and education. Models for fat-free mass as outcome are further adjusted for fat mass.

β coefficients marked in bold are significant at *p<0.05, **p<.01, ***p<.001, respectively.

^1^ Antidepressants with meta-analytical evidence for association with weight gain (i.e., Amitryptilin [ATC [N06AA09](https://www.gelbe-liste.de/atc/Amitriptylin_N06AA09)], Mirtazapin [ATC [N06AX11](https://www.gelbe-liste.de/atc/Mirtazapin_N06AX11)], Paroxetin [ATC [N06AB05](https://www.gelbe-liste.de/atc/Paroxetin_N06AB05)]) ^3,4^.

^2^ Antidepressants (ATC code N06A) with no meta-analytical evidence for associations with weight gain.

^3^ Includes all pharmacological agents classified under ATC code N06A and also includes antidepressants with meta-analytical evidence for association with weight reduction (i.e., Fluoxetin [[N06AB03](https://www.gelbe-liste.de/atc/Fluoxetin_N06AB03)], Bupropion [ATC N06AX12]) ^3,4^. Weight-reduction associated antidepressants were too few to analyse independently (men: n=5; women: n=5).

**Table S4:** Sex-specific associations between MDD recurrence status and obesity-related traits with additional covariates (smoking, alcohol consumption, and physical activity)

|  | **MDD_S_ vs. no MDD**  **β (95%-CI)** | **MDD_R_ vs. no MDD**  **β (95%-CI)** |
| --- | --- | --- |
| **Men** | | |
| BMI (kg/m^2^) | 0.09 (-0.76; 0.93) | 0.24 (-0.51; 0.99) |
| WC (cm) | 0.88 (-1.33; 3.09) | 1.14 (-0.82; 3.09) |
| WHtR | 0.67 (-0.58; 1.92) | 0.25 (-0.85; 1.36) |
| Fat mass (kg) | -0.48 (-2.03; 1.06) | 1.11 (-0.25; 2.47) |
| SAT (L) | **0.95 (0.16; 1.73)*** | **0.89 (0.14; 1.64)*** |
| VAT(L) | 0.65 (-0.02; 1.33) | 0.44 (-0.21; 1.08) |
| Women | | |
| BMI (kg/m^2^) | -**1.06 (-1.98; -0.13)*** | **0.90 (0.23; 1.58)**** |
| WC (cm) | -**3.11 (-5.25; -0.97)**** | **2.28 (0.72; 3.83)**** |
| WHtR | **-2.22 (-3.56; -0.88)**** | **1.23 (0.25; 2.20)*** |
| Fat mass (kg) | -1.32 (-3.07; 0.44) | **2.06 (0.76; 3.36)**** |
| SAT (L) | -0.53 (-1.44; 0.38) | **0.76 (0.04; 1.47)*** |
| VAT(L) | -0.24 (-0.64; 0.15) | **0.39 (0.08; 0.70)**** |

**Note:** β coefficients are derived from sex-stratified linear regression models adjusted for age, education, current depressive symptoms (BDI-2), smoking, alcohol consumption, and physical activity. Models for fat-free mass as outcome are further adjusted for fat mass.

β coefficients marked in bold are significant at *p<0.05, **p<.01, ***p<.001, respectively.

**Supplemental References**

1. WHO. Waist circumference and waist-hip ratio: report of a WHO expert consultation, Geneva, 8-11 December 2008. 2011.

2. Müller HP, Raudies F, Unrath A, Neumann H, Ludolph AC, Kassubek J. Quantification of human body fat tissue percentage by MRI. *NMR in Biomedicine*. 2011;24:17-24.

3. Serretti A, Mandelli L, Laura M. Antidepressants and body weight: a comprehensive review and meta-analysis. *The Journal of clinical psychiatry*. 2010;71:979.

4. Domecq JP, Prutsky G, Leppin A, Sonbol MB, Altayar O, Undavalli C, Wang Z, Elraiyah T, Brito JP, Mauck KF. Drugs commonly associated with weight change: a systematic review and meta-analysis. *The Journal of Clinical Endocrinology & Metabolism*. 2015;100:363-370.
